# The Clinical Differences of Patients With Traumatic Brain Injury in Plateau and Plain Areas

**DOI:** 10.3389/fneur.2022.848944

**Published:** 2022-04-25

**Authors:** Yongxiang Yang, Yuping Peng, Siyi He, Jianping Wu, Qingyun Xie, Yuan Ma

**Affiliations:** ^1^Department of Neurosurgery, General Hospital of Western Theater Command, Chengdu, China; ^2^Department of Neurosurgery, Affiliated Hospital of Southwest Medical University, Luzhou, China; ^3^Department of Cardiovascular Surgery, General Hospital of Western Theater Command, Chengdu, China; ^4^Department of Orthopedic, General Hospital of Western Theater Command, Chengdu, China

**Keywords:** traumatic brain injury, plateau area, plain area, cardiac function, coagulation function

## Abstract

**Objective:**

Traumatic brain injury (TBI) is a leading cause of death and disability, which tends to have a worse clinical recovery if it occurs in plateau areas than in plain areas. To explore the underlying cause of this outcome preliminarily, this retrospective study was conducted to compare the clinical differences of patients with TBI in plateau and plain areas.

**Methods:**

In this study, 32 patients with TBI in plateau areas (altitude ≥ 4,000 m) and 32 in plain areas (altitude ≤ 1,000 m) were recruited according to the inclusion and exclusion criteria from June 2020 to December 2021. The collected data and compared parameters include clinical features, head CT presentations and Marshall classifications, hematology profile, lipid profile, coagulation profile, and multiorgan (cardiac, liver, renal) function within 24 h of hospital admission, as well as the treatment method and final outcome.

**Results:**

There were no obvious differences in demographic characteristics, including gender, age, height, and weight, between patients with TBI in plateau and plain areas (all *P* > 0.05). Compared to patients with TBI in plain areas, the time before hospital admission was longer, heartbeat was slower, systolic blood pressure (SBP) was lower, and hospital stays were longer in patients with TBI in plateau areas (all *P* < 0.05). More importantly, elevated red blood cells (RBCs) count and hemoglobin (HGB) level, enhanced coagulation function, and higher rates of multiorgan (cardiac, liver, and renal) injury were found in patients with TBI in plateau areas (all *P* < 0.05).

**Conclusion:**

Patients with TBI in plateau areas presented with altered clinical characteristics, enhanced coagulation function, and aggravated predisposition toward multiorgan (cardiac, liver, and renal) injury, compared to patients with TBI in plain areas. Future prospective studies are needed to further elucidate the influences of high altitude on the disease course of TBI.

## Introduction

High-altitude areas, such as the Qinghai–Tibet Plateau, are unique districts in western China. With the rapid development of transportation, more and more people traveled to plateau areas for working, recreational, sporting, and other purposes. Correspondingly, the occurrence of accidental injuries at plateau areas increased gradually and raised rapidly by 12.7% (1–3). Accidental injuries caused by traffic accidents at plateau areas had high mortality rates, of which the most serious acute disease was traumatic brain injury (TBI) (2, 3). TBI is a severe life-threatening injury with survivors often suffering from neurological function impairments, which can be even more complicated and tends to have a worse clinical recovery if it occurs in plateau areas than in plain areas (4). The most common TBI type at plateau areas in China is mild-to-moderate closed TBI (mmTBI), whose clinical features have not been well demonstrated so far. Hence, it is of great significance to study the clinical differences of TBI (especially the mmTBI) in plateau areas.

Hypobaric hypoxia environment in plateau areas leads to the reduction of body metabolism and the increasement of water consumption, which further causes multiorgan injury and adds difficulty to the treatment of TBI. More and more studies indicate that TBI not only causes central nervous system (CNS) disorder, but also induces wide-ranging systemic effects, such as altering the biology and function of the heart, lung, liver, kidney, and blood system, which in turn worsen the neurological outcome (5–7). Taking together, the disease course of TBI in plateau areas is multifactorial and may involve multiorgan injury, which has not been sufficiently demonstrated so far. Therefore, it is an urgent need to explore the systemic effects, such as multiorgan injury of patients with TBI in plateau areas. Furthermore, it is important to clarify the prevalence and significance of these effects of TBI in plateau areas as well, as this may contribute to the optimization of treatment and the improvement of recovery.

As far as we know, no previous reports have demonstrated the clinical differences of patients with TBI in plateau and plain areas. Herein, the present study comprehensively investigated parameters, including clinical features, hematology profile, lipid profile, coagulation profile, and multiorgan (cardiac, liver, and renal) function, of patients with TBI in plateau and plain areas within 24 h of hospital admission, as well as the treatment method and final outcome. The purpose of this study was to clarify the clinical characteristics of patients with TBI in plateau areas and provide a theoretical basis for earning more effective treatments and better clinical outcomes.

## Materials and Methods

### Setting

This retrospective study was conducted in China at the General Hospital of Western Theater Command in Chengdu and a tertiary hospital in the plateau area. Clinical data of patients with TBI in plain areas (altitude ≤ 1,000 m) and plateau areas (altitude ≥ 4,000 m) was collected from the former and the latter hospital respectively. Both two hospitals have separate intensive care unit (ICU) beds for trauma and neurosurgery patients. These ICUs have professional intensivist-led teams consisting of critical care physicians and nurses, who are in charge of 24-h medical care. Ethics Committee of the Faculty of The General Hospital of Western Theater Command in Chengdou and the tertiary hospital in the plateau area gave permission for this research. All the studying process in this research was carried out in accordance with the approved guidelines.

### Patients

Patients with TBI in plateau areas (altitude ≥ 4,000 m) and plain areas (altitude ≤ 1,000 m) were recruited from the inpatient service of the tertiary hospital in the plateau area and the General Hospital of Western Theater Command in Chengdu, respectively. The collected data included complete admission and hospitalization records of patients with TBI from June 2020 to December 2021. The inclusion criteria include the following: (1) age ≥ 18 years; (2) discharge diagnosis of TBI (ICD 9 codes 800–801.9, 803–804.9, or 850–854.1); (3) the time duration of patients with TBI in plateau areas (altitude ≥ 4,000 m) is at least 3 months; (4) patients with TBI in plain areas (altitude ≤ 1,000 m) had not been exposed to high altitudes during the past 2 years. Patients with the following situations were excluded: (1) the admission and hospitalization information was incomplete; (2) presence of extracranial injury (such as orthopedic/chest/cardiac/abdominal/pelvis and so on); (3) pre-existing cardiac disease (such as myocardial ischemia/infarction, arrhythmia, heart failure, untreated hypertension, and cardiac pacemaker); (4) combined with liver/renal/lung failure, hematological disease, infection disease, malignancy, pregnancy, and use of anticoagulants or antiplatelet agents.

### Clinical Care for Patients With TBI

Once patients arrived at the emergency department, standard treatments and management were carried out immediately according to the Brain Trauma Foundation Guidelines (8). According to the Glasgow Coma Scale (GCS), patients with TBI were categorized into mild (GCS = 13–15), moderate (GCS = 9–12), and severe (GCS = 3–8) (9). All the patients received comprehensive neurological evaluation and subsequently underwent cranial CT scan. Repeat CT scan was conducted whenever patients presented the indication of clinical deterioration or the sign of intracranial pressure elevation. The brain MRI examination would be performed immediately if the CT scan showed no significant abnormality, but the diagnosis of TBI was suspected. In addition, other routine clinical examinations, including chest X-Ray, abdomen ultrasound, ECG, and laboratory tests, such as hematology, urine and feces analysis, lipid and coagulation profile, and multiorgan (cardiac, liver, and renal) function analysis, were conducted within 12 h after patients were hospitalized.

### Data Collection

All parameters that might be influenced by high attitude according to our existing knowledge and previous literature were analyzed in this study. The collected data included clinical features, head CT presentations and Marshall classifications, hematology profile, lipid profile, coagulation profile, and multiorgan (cardiac, liver, and renal) function within 24 h of hospital admission, as well as the treatment method and final outcome. Clinical features involved baseline demography, altitude and duration of plateau exposure, vital signs of hospital admission, time before hospital admission, key symptoms, and major diagnosis. Based on radiologic findings, TBI was classified into acute subdural hematoma (ASDH), acute epidural hematoma (AEDH), intracerebral hematoma/contusion (ICH/ICC), and traumatic subarachnoid hemorrhage (TSAH). Furthermore, the detailed head CT data, including the Marshall classifications, the degree of brain stem compression, and the midline shift, were recorded as well. Specifically, the hematology profile included hemoglobin (HGB), red blood cells (RBCs) count, total leukocyte count, and total platelet (PLT) count. The lipid profile included total cholesterol (TC), triglyceride (TG), high-density lipoprotein (HDL), and low-density lipoprotein (LDL). The coagulation profile included prothrombin time (PT), thrombin time (TT), activated partial thromboplastin time (APTT), D-dimer (D-D), and fibrinogen (FIB). The cardiac function was evaluated by electrocardiograph (ECG), cardiac ultrasound, and myocardial enzyme consisting of creatine kinase (CK), creatine kinase MB (CK-MB), and lactic dehydrogenase (LDH). The liver function was measured by glutamic oxalacetic transaminase (AST), glutamic-pyruvic transaminase (ALT), total/direct bilirubin (TBIL/DBIL), and albumin. The renal function was evaluated by creatinine, urea nitrogen, and uric acid. Treatment methods included the surgical type and therapeutic drugs. The final outcome was evaluated by hospital stays and neurological function.

### Data Analysis

Measurement data were expressed as mean values ± standard deviations (*M* ± *SD*). Differences between the two groups were analyzed by an unpaired *t*-test with Welch's correction. Relationships between two variables were evaluated using the Spearman rank correlation test. Enumeration data were analyzed by the Chi-squared test. SPSS version 18 software (SPSS Inc., IBM Corp., USA) was used to perform the analysis, and two-tailed *P* < 0.05 was considered statistically significant. Photoshop software (Adobe Software, Inc., USA) was used to draw the figure.

## Results

### Patients Selection

**Figure 1:**
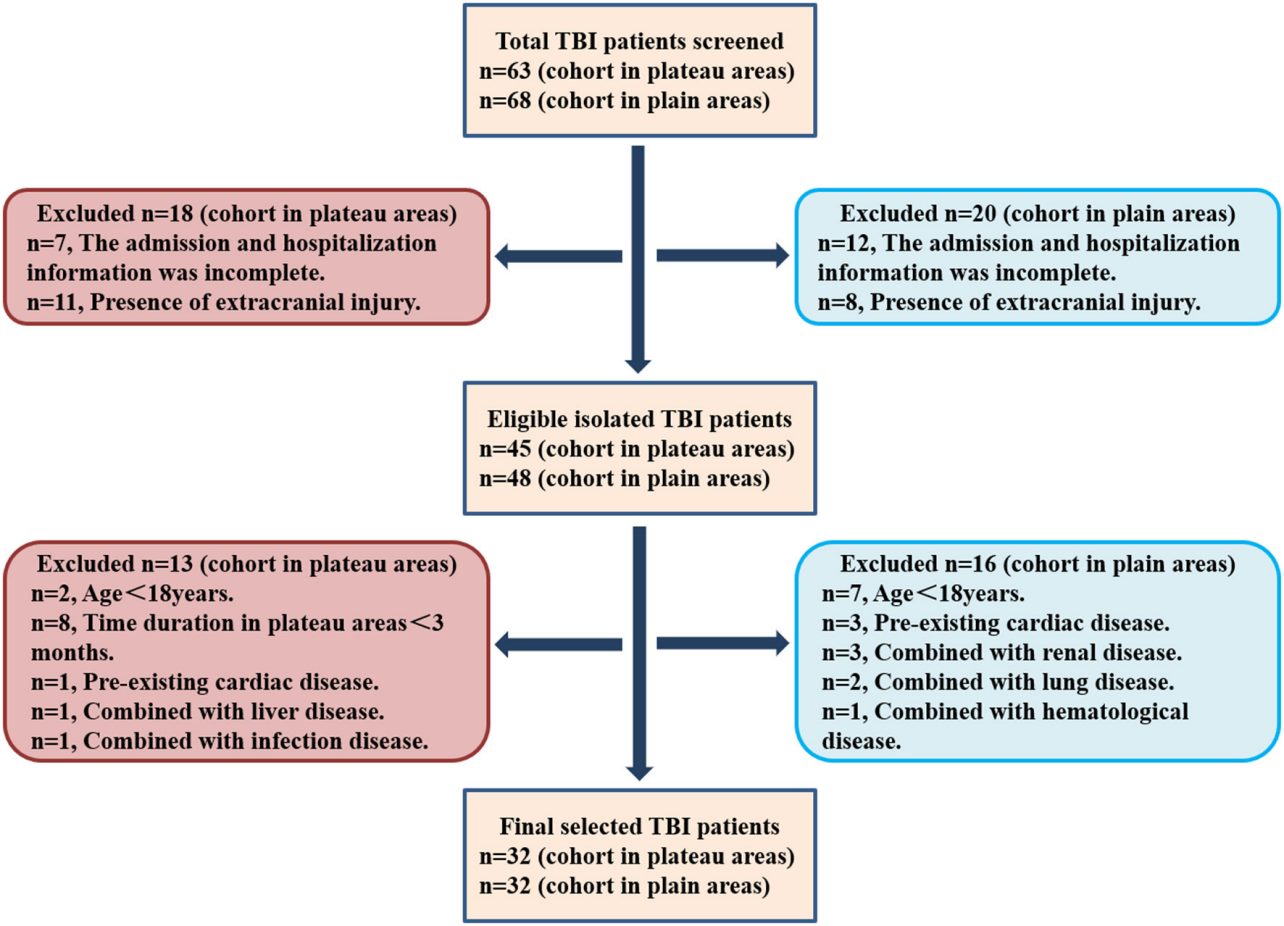
The selection flowchart of patients with traumatic brain injury (TBI) in plateau and plain areas.

All patients with TBI were isolated from the inpatient service of the tertiary hospital in the plateau area and The General Hospital of Western Theater Command in Chengdu from June 2020 to December 2021 using ICD-9 procedural code terminology (ICD 9 codes 800–801.9, 803–804.9, or 850–854.1). Patients were eligible if the diagnosis was isolated TBI, and they were further selected according to the inclusion and exclusion criteria as described in the “Materials and Methods” section. Finally, 32 patients with TBI in plateau areas (altitude≥ 4,000 m) and 32 in plain areas (altitude ≤ 1,000 m) were recruited. The selection flowchart is illustrated in Figure 1.

### Clinical Features of Patients With TBI in Plateau and Plain Areas Had Significant Differences

Patients with TBI in plateau and plain areas had similar gender, age, height, and weight distribution, indicating the demographic baseline of these two groups was well balanced. The mean altitude of residence and duration at plateau areas was 4,089.4 ± 369.3 m and 195.8 ± 84.6 days. The time before hospital admission since TBI occurred was obviously longer in patients at plateau areas than at plain areas (92.6 ± 132.6 h vs. 30.9 ± 50.1 h, *P* = 0.017). Patients with TBI in plateau and plain areas had similar mean GCS scores and severity of illness, which also indicated the most common TBI type of them was mild-to-moderate TBI (mmTBI) (96.9 vs. 100%, *P* = 0.559). As for the vital signs at hospital admission, patients with TBI in plateau areas had a slower heartbeat, lower systolic blood pressure (SBP), and faster breathing than in plain areas (*P* = 0.032, < 0.001, and 0.009, respectively), but the body temperature and diastolic blood pressure (DBP) was similar. Detailed data are shown in Table 1.

**Table 1:** Demographic and clinical characteristics of patients with traumatic brain injury (TBI) in plateau and plain areas.

**Variable**	**TBI in plateau**	**TBI in plain**	**T/χ^2^ value**	***P*** **value**
Gender (*n*, %)			*n*.a	*n*.a
Male	32 (100)	32 (100)		
Female	0 (0)	0 (0)		
Age (y)	25.7 ± 3.5	24.3 ± 3.8	1.549	0.126
Height (cm)	173.4 ± 6.1	171.9 ± 5.9	1.006	0.318
Weight (kg)	69.9 ± 10.4	72.6 ± 6.4	−1.245	0.218
Altitude of residence (m)	4,089.4 ± 369.3	633.4 ± 97.4	61.855	<0.001*
Duration at plateau (d)	195.8 ± 84.6	0	13.09	<0.001*
Time before admission (h)	92.6 ± 132.6	30.9 ± 50.1	2.461	0.017*
GCS	12.2 ± 1.8	12.4 ± 1.2	−0.485	0.63
TBI severity (*n*, %)			1.164	0.559
Mild	14 (43.8)	16 (50)		
Moderate	17 (53.1)	16 (50)		
Severe	1 (3.1)	0 (0)		
Vital signs				
Body temperature (°C)	36.8 ± 0.3	36.8 ± 0.6	−0.297	0.768
Heartbeat (times/min)	72.7 ± 14.4	79.9 ± 12.1	−2.198	0.032*
SBP (mmHg)	110.6 ± 6.8	124.4 ± 10.8	−0.612	<0.001*
DBP (mmHg)	73.4 ± 5.1	73.8 ± 8.0	−0.186	0.853
Breathing (times/min)	21.2 ± 2.8	19.7 ± 1.6	2.703	0.009*

GCS, Glasgow Coma Scale; SBP, systolic blood pressure; DBP, diastolic blood pressure. P < 0.05 represents statistically significant (marked with ^*^ and bold).

### Head CT Presentations of Patients With TBI in Plateau and Plain Areas

As shown in Table 2, the majority of patients with TBI in plateau areas were AEDH (37.5%) and ASDH (34.4%), whereas those in plain areas were ICH/ICC (50%), which difference was significant (*P* = 0.027). Moreover, the comparison of detailed head CT presentations, including the Marshall classifications, the degree of brain stem compression, and the midline shift between these two groups, were performed as well. Though the rate of basal cisterns compression, the degree of midline shift, and Marshall classification were higher in patients with TBI in plateau areas than those in plain areas, these differences were not statistically significant. In brief, the above data indicated that patients with TBI in plateau areas and plain areas presented with different CT diagnoses, but had similar brain edema extent. These results were consistent with the GCS score and TBI severity described previously.

**Table 2:** Head CT presentations of patients with TBI in plateau and plain areas.

**Variable**	**TBI in plateau**	**TBI in plain**	**χ^2^value**	***P*** **value**
Major CT diagnosis (*n*, %)			9.162	0.027*
ASDH	11 (34.4)	8 (25)		
AEDH	12 (37.5)	4 (12.5)		
ICH/ICC	6 (18.8)	16 (50)		
TSAH	3 (9.3)	4 (12.5)		
Basal cisterns (*n*, %)			0.721	0.396
Normal	22 (68.8)	25 (78.1)		
Compressed	10 (31.2)	7 (21.9)		
Obliterated	0	0		
Midline shift (*n*, %)			1.269	0.736
0 mm	15 (46.9)	16 (50)		
1–5 mm	13 (40.6)	14 (43.8)		
5–10 mm	3 (9.4)	2 (6.2)		
> 10 mm	1 (3.1)	0 (0)		
Marshall CT classification (*n*, %)			1.715	0.788
I	1 (3.1)	2 (6.2)		
II	21 (65.8)	23 (71.9)		
III	6 (18.7)	5 (15.7)		
IV	3 (9.3)	2 (6.2)		
V or VI	1 (3.1)	0 (0)		

CT, computerized tomography; ASDH, acute subdural hematoma; AEDH, acute epidural hematoma; ICH/ICC, intracerebral hematoma/contusion; TSAH, traumatic subarachnoid hemorrhage. P < 0.05 represents statistically significant (marked with ^*^ and bold).

### Patients With TBI in Plateau Areas Presented With Exaggerated Hypercoagulative State

As shown in Table 3, HGB level, RBCs count, hematocrit (HCT) was obviously higher (both *P* < 0.001), and PLT count was considerably lower (*P* = 0.028) in patients with TBI in plateau areas than those in plain areas, but the total leukocyte count of them was similar. And, the lipid profile analysis indicated that patients with TBI in plateau areas had lower levels of TC and TG (*P* < 0.001 and *P* = 0.021), but had higher levels of LDL (*P* = 0.001) than those in plain areas. At last, it was worth noting that all coagulation indicators were obviously different between patients with TBI in plateau areas and plain areas. Specifically, patients with TBI in plateau areas presented with considerably longer PT, APTT, and TT (*P* =0.001, <0.001, and <0.001, respectively), as well as obviously lower levels of FIB and higher levels of D-D (both *P* < 0.001) than in plain areas. Overall, the above data showed that patients with TBI in plateau areas presented with an exaggerated hypercoagulative state.

**Table 3:** Hematology/lipid/coagulation profile of patients with TBI in plateau and plain areas.

**Variable**	**TBI in plateau**	**TBI in plain**	***T* value**	***P*** **value**
Hematology				
HGB (mg/dL)	188.7 ± 35.0	144.0 ± 18.3	6.399	<0.001*
RBCs (10^12^/L)	6.3 ± 0.8	4.8 ± 0.6	7.787	<0.001*
HCT (%)	52.8 ± 9.8	42.9 ± 5.6	5.028	<0.001*
PLT (10^9^/L)	171.5 ± 54.2	197.5 ± 36.2	−2.252	**0.028**
Total leukocytes (10^9^/L)	14.9 ± 8.1	15.1 ± 5.6	−0.096	0.924
Lipid profile (mg/dL)				
Total cholesterol (mmol/L)	3.4 ± 0.8	4.3 ± 0.9	−4.559	<0.001*
Triglycerides (mmol/L)	0.9 ± 0.4	1.3 ± 0.8	−2.369	0.021*
HDL (mmol/L)	1.3 ± 1.6	1.2 ± 0.3	0.247	0.806
LDL (mmol/L)	1.9 ± 0.6	2.6 ± 0.9	−3.393	0.001*
Coagulation profile				
PT (s)	22.1 ± 16.9	11.7 ± 1.9	3.448	0.001*
APTT (s)	35.5 ± 11.4	26.8 ± 3.9	4.098	<0.001*
TT (s)	27.0 ± 6.0	18.3 ± 3.0	7.268	<0.001*
FIB (mg/dL)	1.6 ± 0.5	2.3 ± 0.5	−5.546	<0.001*
D-D (mg/L)	7.9 ± 11.3	0.5 ± 1.3	3.68	<0.001*

HGB, hemoglobin; RBCs, red blood cells; HCT, hematocrit; PLT, platelet; HDL, high-density lipoprotein; LDL, lower-density lipoprotein; PT, prothrombin time; APTT, activated partial thromboplastin time; TT, thrombin time; FIB, fibrinogen; DD, D-dimer. P < 0.05 represents statistically significant (marked with ^*^ and bold).

### Correlation Between Basic Coagulation Parameters and RBCs Indexes in Patients With TBI

As previous data indicated that patients with TBI in plateau areas presented with exaggerated hypercoagulative state, the association between basic coagulation parameters (APTT, PT, TT, FIB, and PLT) and RBCs indexes (HGB and HCT) were further analyzed. Results in Table 4 showed that PT was negatively correlated with HGB level (*r* = −0.401, *P* = 0.023) and positively correlated with HCT in patients with TBI in plateau areas (*r* = 0.428, *P* = 0.015), but APTT, TT, FIB, and PLT had no correlation with HGB and HCT. Additionally, positive correlations were found between TT and HGB, TT and HCT, PLT and HGB, and PLT and HCT in patients with TBI in plain areas (*r* = 0.477 *P* = 0.006, *r* = 0.383 *P* = 0.031, *r* = 0.341 *P* = 0.047, and *r* = 0.363 *P* = 0.041, respectively), but PT, APTT, and FIB had no correlation with HGB and HCT. Next, the association between basic coagulation parameters APTT, PT, TT and PLT, D-D, and FIB were analyzed as well. As shown in Table 5, PLT was negatively correlated with PT in patients with TBI in plateau areas (*r* = −0.283, *P* = 0.046), but they had no correlation in those in plain areas. Additionally, FIB was negatively correlated with D-D in patients with TBI in plain areas (*r* = −0.535, *P* = 0.002), but they had a positive correlation in those in plateau areas (*r* = 0.563, *P* = 0.001).

**Table 4:** Correlation between coagulation parameters and red blood cells (RBCs) indexes in patients with TBI.

	**TBI in plateau areas**		**TBI in plain areas**	
	**HGB** **(mg/dL)** **(r, p)**	**HCT** **(%)** **(r, p)**	**HGB** **(mg/dL)** **(r, p)**	**HCT** **(%)** **(r, p)**
PT (s)	−0.401, 0.023*	0.428, 0.015*	−0.208, 0.254	−0.048, 0.796
APTT (s)	−0.002, 0.993	−0.010, 0.956	−0.043, 0.815	0.048, 0.794
TT (s)	0.131, 0.474	0.128, 0.486	0.477, 0.006*	0.383, 0.031*
FIB (mg/dL)	−0.315, 0.079	−0.276, 0.127	−0.133, 0.468	−0.088, 0.634
PLT (10^9^/L)	−0.203, 0.266	−0.204, 0.263	0.341, 0.047*	0.363, 0.041*

HGB, hemoglobin; HCT, hematocrit; PT, prothrombin time; APTT, activated partial thromboplastin time; TT, thrombin time; FIB, fibrinogen; PLT, platelet. P < 0.05 represents statistically significant (marked with ^*^ and bold).

**Table 5:** Correlation between coagulation parameters and RBCs indexes in patients with TBI.

	**TBI in plateau areas**		**TBI in plain areas**	
	**PLT (10^9^/L)** **(r, p)**	**DD (mg/L)** **(r, p)**	**PLT (10^9^/L)** **(r, p)**	**DD (mg/L)** **(r, p)**
PT (s)	−0.283, 0.046*	*n*.a	−0.241, 0.184	*n*.a
APTT (s)	−0.095, 0.606	*n*.a	0.058, 0.752	*n*.a
TT (s)	−0.181, 0.321	*n*.a	−0.002, 0.990	*n*.a
FIB (mg/dL)	*n*.a	−0.535, 0.002*	*n*.a	0.563, 0.001*

PT, prothrombin time; APTT, activated partial thromboplastin time; TT, thrombin time; FIB, fibrinogen; PLT, platelet; DD, D-dimer. P < 0.05 represents statistically significant (marked with ^*^ and bold).

### Patients With TBI in Plateau Areas Had a Higher Rate of Cardiac Dysfunction

Table 6 demonstrates cardiac function evaluation, including myocardial enzyme, ECG, and echocardiogram. First, the level of CK, CK-MB, and LDH were obviously higher in patients with TBI in plateau areas than those in plain areas (*P* < 0.001, =0.006, and < 0.001, respectively). Second, arrhythmia was considerably more frequent among patients with TBI in plain areas than at plateau areas (46.9 vs. 6.2%, *P* < 0.001). Additionally, the ST-T alteration was more frequent among patients with TBI in plateau areas than those in plain areas (50 vs. 18.6%, *P* = 0.035), of which the most common one was ST elevation (25%). Furthermore, patients with TBI in plateau areas had higher rates of reduced left ventricular ejection fraction (LVEF) (<50%) and regional wall motion abnormality (RWMA) than at plain areas (31.2 vs. 6.2%, *P* = 0.01, and 34.4 vs. 9.4%, *P* = 0.043). Taking together, these data indicated that patients with TBI in plateau areas had higher rates of cardiac dysfunction.

**Table 6:** Cardiac function of patients with TBI in plateau and plain areas.

**Variable**	**TBI in plateau**	**TBI in plain**	**T/χ^2^ value**	***P*** **value**
Myocardial enzyme				
CK (mg/dL)	596.1 ± 660.9	99.5 ± 57.8	4.234	<0.001*
CK-MB (mg/dL)	31.8 ± 19.6	20.6 ± 10.9	2.827	0.006*
LDH (mg/dL)	235.4 ± 126.2	142.0 ± 35.6	4.029	<0.001*
Electrocardiograph-rhythm (*n*, %)			13.537	<0.001*
Sinus rhythm	30 (93.8)	17 (53.1)		
Arrhythmia	2 (6.2)	15 (46.9)		
Electrocardiograph-ST-T (*n*, %)			8.635	0.035*
Normal ST-T	16 (50)	26 (81.4)		
ST depression	4 (12.5)	3 (6.2)		
ST elevation	8 (25)	1 (3.1)		
Inverted T wave	4 (12.5)	2 (6.3)		
Cardiac ultrasound (*n*, %)			6.564	0.01*
Normal LVEF (LVEF ≥ 50%)	22 (68.8)	30 (93.8)		
Low LVEF (LVEF <50%)	10 (31.2)	2 (6.2)		
Cardiac ultrasound-RWMA (*n*, %)				
RWMA-normal	21 (65.6)	29 (90.6)	6.28	0.043*
RWMA-mild hypokinesis	9 (28.2)	3 (9.4)		
RWMA-severe hypokinesis	2 (6.2)	0 (0)		

CK, creatine kinase; CK-MB, creatine kinase isoenzyme; LDH, lactic dehydrogenase; LVEF, left ventricular ejection fraction; RWMA, regional wall motion abnormality. P < 0.05 represents statistically significant (marked with ^*^ and bold).

### Patients With TBI in Plateau Areas Had More Predisposition Toward Liver/Renal Function Injury

Liver and renal function evaluation is displayed in Table 7. Patients with TBI in plateau areas presented with higher levels of ALT, TBIL, and lower levels of AST than at plain areas (*P* = 0.046, 0.001, and 0.038, respectively). Moreover, liver injury was more frequent among patients with TBI in plateau areas than at plain areas (56.3 vs. 31.3%, *P* = 0.044). The levels of creatinine and urea nitrogen were higher in patients with TBI in plateau areas than at plain areas (both *P* < 0.001). However, the percentages of renal injury had no significant differences between them (25 vs. 12.5%, *P* = 0.2). In brief, patients with TBI in plateau areas had more predisposition toward liver/renal function injury.

**Table 7:** Liver and renal function of patients with TBI in plateau and plain areas.

**Variable**	**TBI in plateau**	**TBI in plain**	**T/χ^2^ value**	***P*** **value**
Liver function				
AST (U/L)	34.8 ± 19.8	47.3 ± 26.9	−2.115	0.038*
ALT (U/L)	47.7 ± 22.5	35.6 ± 25.0	2.032	0.046*
TBIL (umol/L)	28.5 ± 14.9	17.5 ± 9.3	3.55	0.001*
DBIL (umol/L)	6.4 ± 3.3	5.7 ± 3.1	0.847	0.4
Albumin (g/L)	42.4 ± 5.9	44.1 ± 3.4	−1.443	0.154
Liver injury (*n*, %)			4.063	0.044*
With	18 (56.3)	10 (31.3)		
Without	14 (43.7)	22 (68.7)		
Renal function				
Creatinine (umol/L)	92.7 ± 18.5	69.3 ± 15.1	5.54	<0.001*
Urea nitrogen (mmol/L)	7.4 ± 1.9	4.6 ± 2.1	5.63	<0.001*
Uric acid (umol/L)	393.2 ± 84.2	340.5 ± 91.0	2.4	0.019
Renal injury (*n*, %)			1.641	0.2
With	8 (25)	4 (12.5)		
Without	24 (75)	28 (87.5)		

AST, glutamic oxalacetic transaminase; ALT, glutamic-pyruvic transaminase; TBIL, total bilirubin; DBIL, direct bilirubin. P < 0.05 represents statistically significant (marked with ^*^ and bold).

### Patients With TBI in Plateau Areas Had Longer Hospital Stays and Worse Outcome

At last, differences in the treatments and outcomes among patients with TBI in two areas were analyzed. As shown in Table 8, the rate of craniotomy in patients with TBI in plateau areas and plain areas was similar (37.5 vs. 28.1%, *P* = 0.044). Additionally, all the craniotomy manner in the two groups was the removal of hemorrhage. The percentage of neurological dysfunction post-discharge was higher in patients with TBI in plateau than those in plain areas (31.3 vs. 9.4%, *P* = 0.03). Furthermore, the hospital stays of patients with TBI in plateau areas was longer than those in plain areas (*P* < 0.001). These findings showed that patients with TBI in plateau areas had longer hospital stays and worse outcomes.

**Table 8:** Treatment and outcome in patients with TBI at the plateau and plain areas.

**Variable**	**TBI in plateau**	**TBI in plain**	**T/χ^2^ value**	***P*** **value**
Craniotomy (*n*, %)			0.638	0.424
With	12 (37.5)	9 (28.1)		
Without	20 (62.5)	23 (71.9)		
Craniotomy manner (*n*, %)			*n*.a	*n*.a
Removal of hemorrhage	12 (100)	10 (100)		
Decompressive •craniectomy	0	0		
Neurological dysfunction (*n*, %)			4.73	0.03*
With	10 (31.3)	3 (9.4)		
Without	22 (68.7)	29 (90.6)		
Hospital stays (d)	12.9 ± 3.2	9.9 ± 2.1		<0.001*

P < 0.05 represents statistically significant (marked with ^*^ and bold).

## Discussion

This retrospective study comprehensively investigated the clinical differences of patients with TBI in plateau and plain areas. Major results were as follows: (1) patients with TBI in plateau areas had a longer time before hospital admission and slower heartbeat, lower SBP, and faster breathing at hospital admission. (2) The majority of patients with TBI in plateau areas were AEDH and ASDH, while in plain areas were ICH/ICC. (3) The hypercoagulative state was obviously exaggerated in patients with TBI in plateau areas. (4) Patients with TBI in plateau areas had a higher rate of cardiac dysfunction. (5) Patients with TBI in plateau areas had more predisposition toward liver/renal function impairment. (6) Patients with TBI in plateau areas had longer hospital stays and worse outcomes. These findings are significant for clarifying the clinical characteristics of patients with TBI in plateau areas.

Nowadays, millions of people travel to high-altitude areas every year. The increased number of people making a journey to high altitudes might further bring about the rise in morbidity of TBI in plateau areas (10). Previous studies reported that mmTBI was the most common TBI type in plateau areas in China. Similarly, nearly all the recruited patients with TBI in plateau and plain areas in this research were mmTBI. Meanwhile, the GCS score and head CT Marshall classification of patients with TBI in the two areas had no differences. These observations indicated that the two groups were homogeneous with each other on the TBI severity baseline. However, the subsequent analysis found the percentage of neurological dysfunction post-discharge was higher and the hospital stays were longer in patients with TBI in plateau areas. In a word, these findings showed that patients with TBI in plateau areas had worse outcomes. The cause of this outcome might be multifactorial. First, hypoxia in plateau areas is a critical factor aggravating the condition of TBI and influencing the prognosis of TBI (10, 11). Second, the harsh geographical environment in plateau areas makes great difficulty for the timely transportation and treatment of patients with TBI, as this study found the time before hospital admission since TBI occurring was obviously longer in patients at plateau areas. Moreover, higher rates of multiorgan (cardiac, liver, renal) injury in patients with TBI at plateau areas might be another important factor, which was found in this study and will be extensively discussed in the following section.

Traumatic brain injury is a complex and systemic condition, it not only leads to the impairment of neurological function, but also cause an extensive pathophysiological effect on peripheral organs, such as the heart, lung, liver, and kidney (5–7). Lots of pieces of evidence have demonstrated that TBI can cause cardiac dysfunction, including arrhythmia, ST-T alteration, elevated myocardial enzyme, decreased LVEF, and RWMA (6, 12–14). And, high-altitude alone is an important factor that can cause cardiac morphology alterations and function impairments (15, 16). Based on these reports, the speculation that exposure to plateau areas may aggravate cardiac dysfunction can be made. As expected, this study demonstrated that patients with TBI in plateau areas had obviously higher levels of CK, CK-MB, LDH, and higher rates of reduced LVEF (<50%) and RWMA than those in plain areas. In addition, the ST-T alteration was more frequent in patients with TBI in plateau areas, of which the most common one was ST elevation. These findings strongly indicated that patients with TBI in plateau areas had a higher rate of cardiac dysfunction. Moreover, previous research reported that acute plateau exposure caused an increase in heart rate and a decrease of SBP, but chronic plateau exposure caused the opposite effect (15). This study showed patients with TBI in plateau areas had a slower heartbeat, which could be explained by the fact that time duration in plateau areas was at least 3 months, and the mean value was 195.8 ± 84.6 days. However, it is difficult to explain lower SBP in patients with TBI in plateau areas. Hence, more studies are needed to explore this confusion likewise.

Apart from cardiac dysfunction, liver/renal function impairments were also observed in this study. For one thing, the liver injury was more frequent in patients with TBI in plateau areas, which could be explained by a higher level of ALT and TBIL. For another, the level of creatinine and urea nitrogen was higher in patients with TBI in plateau areas, and the percentage of renal injury was higher but without significance. Previous studies have demonstrated that multiple systemic and local changes occur following TBI, including liver and renal injury (5, 7, 17–20). Concerning the liver, TBI can modulate the synthesis of acute-phase proteins by hepatocytes and exert the neuro-innate immune response, which further induces acute liver injury with increased expression of oxidative stress markers and mitochondrial dysfunction within hepatocytes (5, 17, 18). As for the kidney, TBI may cause acute kidney injury through increased sympathetic nervous system activity and plasma catecholamines, systolic hypertension, and hypovolemia by the brain–kidney crosstalk (7, 19–21). What's more, high-altitude alone is a critical factor leading to liver and renal injury as well. On the one hand, staying at plateau areas caused liver dysfunction through endoplasmic reticulum stress and subsequent apoptosis, decreased expression content of hepatic microsomal enzyme, and might also be related to the enzyme's activity changes (22, 23). On the other hand, exposure to high-altitude exerted significant effects on kidney injury by the alteration of cilia length and function which was resulted from the renal hypoperfusion caused by the chronic hypobaric hypoxic environment (24, 25). Taking together, TBI and high-altitude jointly lead to the liver and renal injury through a series of complicated mechanisms, which may provide basics for treatment and should be further explored.

Another important finding was the exaggerated hypercoagulative state of patients with TBI in plateau areas. First, HGB level, RBCs count, HCT was obviously higher and the PLT count was considerably lower in patients with TBI plateau areas than those in plain areas. RBCs play a clear role in the adaptation to hypoxia in plateau areas because of their important role in systemic oxygen transport and delivery (26). It was reported that high-altitude exposure caused the elevation of HGB and HCT levels, which could improve the oxygen-carrying capacity of RBCs but augment the clinical risk of thrombosis (27, 28). PLT is another factor playing a key role in thrombogenesis. Previous studies showed different effects of high-altitude exposure on PLT count (29). The result of this study is consistent with most of the other studies indicating that PLT counts were lower in plateau areas than in plain areas (23, 29–31). Second, patients with TBI in plateau areas had considerably longer PT, APTT, and TT, as well as lower levels of FIB and higher levels of D-D. These results were consistent with other studies which also found PT and APTT were notably prolonged at plateau areas (29, 30, 32), indicating the augmentation of bleeding tendency. However, it seems to be contradictory to the conclusion that high-altitude is a risk factor for thrombosis, as high-altitude can activate the coagulation cascade and lead to increased thrombosis (32). In addition, this study found lower levels of FIB and higher levels of D-D, which was contrary to other studies as well (23, 29, 30). Although the alterations of basic coagulation parameters (APTT, PT, TT, FIB, and PLT) and RBCs indexes (HGB and HCT) were illustrated, the interrelation of these factors was still unclear. Hence, further analysis was performed to gain insights into the relationship among major factors which play critical roles in the coagulation of patients with TBI in plateau areas than those in plain areas. Results showed that PT was negatively correlated with HGB level and positively correlated with HCT in patients with TBI in plateau areas, but APTT, TT, FIB, and PLT had no correlation with HGB and HCT. Additionally, PLT was negatively correlated with PT in patients with TBI in plateau areas, but they had no correlation at plain areas. Moreover, FIB was negatively correlated with D-D in patients with TBI in plain areas, but they had a positive correlation at plateau areas. These results could be explained by the influence of high-altitude exposure and TBI condition. Firstly, high-altitude induced the compensatory hyperplasia of RBCs and the elevation of blood viscosity, which might accelerate the consumption of coagulation factors and ultimately lead to prolonged PT and APTT. Secondly, TBI alone could alter the intricate balance between the mechanism of bleeding and thrombosis, which presented with a range of changes affecting PLT counts and function, procoagulant or anticoagulant factors, fibrinolysis, and interactions among coagulation parameters (33–35). It is worth noting that correlations between the included variables were weak, which might be an epiphenomenon and produced by high-altitude or TBI itself and had little clinical or physiological significance. Herein, it might be difficult to make precise conclusions among the erythrocytes and the basic parameters of coagulation with these results. Additionally, further studies are needed to analyze the influence of high altitude and TBI on the coagulation function and investigate the potential mechanisms.

Conventionally, some limitations in this study should be considered. The first limitation is this study is an observational and retrospective one without intervention and follow-up visits. Second, the number of subjects in this study is relatively small and recruited patients were only men because the particular geographical environment in plateau areas restricted the population size. Finally, all patients with TBI recruited in this study are mmTBI with the purpose to make patients in plateau and plain areas had similar equal severity of illness. However, these limitations also contributed to the homogeneity of patients with TBI in two areas in this study.

In conclusion, this retrospective study demonstrated that patients with TBI in plateau areas presented with altered clinical characteristics, enhanced coagulation function, and aggravated predisposition toward multiorgan (cardiac, liver, renal) injury, as compared to patients with TBI in plain areas. These findings suggest that TBI can affect peripheral organ function, which needs to be considered when planning therapeutic treatments for TBI-polytrauma. Additionally, new therapeutic approaches that not only can reduce neurological deficits but also can address the multiorgan dysfunction induced by TBI must be developed. In addition, future prospective studies are needed to further elucidate the influences of high altitude on the disease course of TBI and explore the potential mechanisms.

## Data Availability Statement

The raw data supporting the conclusions of this article will be made available by the authors, without undue reservation.

## Author Contributions

YM and QX designed the study and guided the writing of this article. YY, YP, and SH conducted the study and wrote the manuscript. JW acquired and analyzed the data. All authors included in this article agreed with the final manuscript.

## Conflict of Interest

The authors declare that the research was conducted in the absence of any commercial or financial relationships that could be construed as a potential conflict of interest.

## Publisher's Note

All claims expressed in this article are solely those of the authors and do not necessarily represent those of their affiliated organizations, or those of the publisher, the editors and the reviewers. Any product that may be evaluated in this article, or claim that may be made by its manufacturer, is not guaranteed or endorsed by the publisher.
